# Role of *Ulva lactuca* Extract in Alleviation of Salinity Stress on Wheat Seedlings

**DOI:** 10.1155/2014/847290

**Published:** 2014-11-10

**Authors:** Wael M. Ibrahim, Refaat M. Ali, Khaulood A. Hemida, Makram A. Sayed

**Affiliations:** ^1^Botany Department, Faculty of Science, Fayoum University, Fayoum, Egypt; ^2^Plant Protection Department, Faculty of Agriculture, Fayoum University, Fayoum, Egypt

## Abstract

Seaweeds are potentially excellent sources of highly bioactive materials that could represent useful leads in the alleviation of salinity stress. The effects of presoaking wheat grains in water extract of *Ulva lactuca* on growth, some enzymatic activities, and protein pattern of salinized plants were investigated in this study. Algal presoaking of grains demonstrated a highly significant enhancement in the percentage of seed germination and growth parameters. The activity of superoxide dismutase (SOD) and catalase (CAT) increased with increasing the algal extract concentration while activity of ascorbate peroxidase (APX) and glutathione reductase (GR) was decreased with increasing concentration of algal extract more than 1% (w/v). The protein pattern of wheat seedling showed 12 newly formed bands as result of algal extract treatments compared with control. The bioactive components in *U. lactuca* extract such as ascorbic acid, betaine, glutathione, and proline could potentially participate in the alleviation of salinity stress. Therefore, algal presoaking is proved to be an effective technique to improve the growth of wheat seedlings under salt stress conditions.

## 1. Introduction

Salinity is one of the major abiotic stresses which limit the yield of major crops [1–3]. It was estimated that up to 20% of irrigated lands in the world are affected by different levels of salinity and sodium content [4]. Salinity stress limits plant growth by adversely affecting various physiological and biochemical processes like photosynthesis, antioxidant phenomena, and nitrogen metabolism [5–7].

Seed germination is an important and critical development phase in the life cycle of plants [8] and is a major limiting factor for establishment plants under saline conditions [9]. Salt stress affects germination percentage, germination rate, and seedling growth in different ways depending on plant species [10, 11].

Seaweeds are macroscopic algae, growing in intertidal and subtidal regions of the sea, and serve as an excellent source of food, fodder, fertilizer, and industrial raw material [12]. The use of marine macroalgae to stimulate the crop production has a long tradition in coastal areas all over the world. Recently, bioactive substances extracted from marine algae are used in agricultural and horticultural crops as biofertilizers to improve their yield and quality and moreover to reduce the negative environmental impact [13].

Seaweeds provide an excellent source of bioactive compounds such as essential fatty acids, vitamins, amino acids, minerals, and growth promoting substances [14, 15], also they have been reported to stimulate the growth and yield of plants [14], enhance antioxidant properties, and develop tolerance to salinity stress [16].

Although a lot of study on seaweeds has been reported on their taxonomy, distribution, photochemistry, and antibacterial activities, a little work has been done on the influence of their extract on the growth of wheat plant under salt stress conditions. Therefore this study was planned to determine the effect of* Ulva lactuca* extract on wheat grain subjected to salt stress during germination and seedling growth through change in nonenzymatic, enzymatic, antioxidant, and protein patterns.

## 2. Material and Method

### 2.1. Plant Materials

Grains of wheat (*Triticum aestivum* L.) were obtained from Horticultural Research Institute, Agricultural Research Center, Ministry of Agriculture, Giza, Egypt.

**Figure 1:**
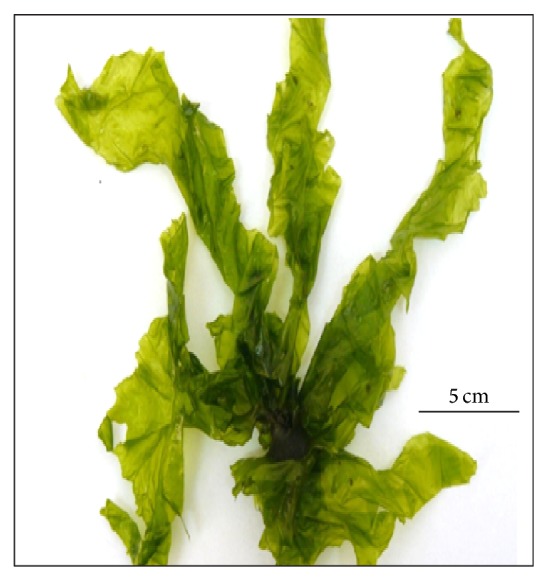
Collected marine macroalgal species* Ulva lactuca.*

Green alga* Ulva lactuca* was collected from Lake Qarun (Fayoum governorate, Egypt) during summer season 2012. The collected algal species (Figure 1) was identified according to Nasr [17] and Jha et al. [18]. The alga was washed with tap water and then with distilled water several times to remove impurities. The fresh seaweed sample was homogenised in distilled water (1 : 1 w/v) at ambient temperature, filtered, and stored. The liquid extract was taken as 100% concentration. From the latter extract, different concentrations (1%, 5%, and 10%) were prepared using distilled water.

### 2.2. Experimental Design

A homogenous lot of grains of wheat plant were selected for uniformity of size, shape, and viability. Before germinating, the grains were surface sterilized by soaking for 3 minutes in 2.5% sodium hypochlorite solution, after which they were washed several times with distilled water. The sterilized grains were presoaked in distilled water (control) and different concentrations of algal extract (1, 5, and 10%) for 12 hours. Thereafter the grains were allowed to drain for one hour. The grains were transferred to sterile petri dishes containing two sheets of Whitman number 1 filter paper moistened with 15 mL of different concentrations of NaCl solutions (0, 50, 100, 150, 200, and 250 mM). Each petri dish contained 20 grains and each treatment was replicated 3 times. The grains were allowed to germinate at 25°C in the darkness and 2 mL of NaCl solutions was added to each petri dish on the third day of the germination. At the end of the experimental period (7 days), the germination percentage, seedlings—fresh and dry matter, some metabolites, and some enzymes activities were recorded in addition to protein patterns.

### 2.3. Electrophoretic Analysis

The extraction of seedling proteins was carried out according to Polar [19]. Fresh seedlings (3 : 1 buffer volume : fresh weight) were homogenized in ice cold 250 mM Tris-sucrose buffer (pH 7.2) in a chilled pestle. The homogenate was filtered through cheesecloth and centrifuged at 12,500 r.p.m for 20 min at 4C°. Protein extract (400 *μ*L) was added to 100 *μ*L SDS 10% and 25 *μ*L p-mercaptoethanol (P-ME), the mixture was heated in boiling bath for 4 min and was cooled down to the room temperature, and then bromo phenol blue (2 drops) was added. The samples were kept in deep freezer until used.

Polyacrylamide gel electrophoresis (PAGE) in the presence of sodium dodecyl sulfate (SDS) was used for determining the molecular weight of the extracted proteins [20]. Electrophoresis was carried out at 150 volt/hours. The gel was analyzed by TotalLab 1D gel analyzer (TotalLab Company, England). The analysis of protein in the control bands is considered as 100% and the protein content in all other treatments estimated as a percent content according to the content of the control bands.

### 2.4. Enzyme Activity Assay

Samples of plant tissues (0.5 g) were homogenized in ice cold 0.1 M phosphate buffer (pH = 7.5) containing 0.5 mM EDTA. Each homogenate was centrifuged at 4°C for 15 min at 15000 g. The supernatant was used for enzyme activity assay [21]. SOD activity was estimated according to Gupta et al. [22], CAT activity was measured according to Aebi [23], APX activity was measured according to Yoshimura et al. [24], and GR activity was assayed according to Sairam et al. [25].

### 2.5. Determination of Glutathione

Glutathione was extracted by grinding 0.5 g of plant tissues in 1% picric acid (w/v) under cold condition. After centrifugation at 10,000 g for 10 min, the supernatant was collected immediately for assay [26].

### 2.6. Estimation of Ascorbic Acid

The total ascorbic acid content was estimated using Folin phenol reagent [27].

### 2.7. Algal Biomass Analysis

Soluble sugars were extracted from algal material according to the method adopted by Upmeyer and Koller [28]. The residue which remained after the extraction of soluble sugars was hydrolyzed with 0.2 N H_2_SO_4_ by reflux in boiling water bath for one hour [29]. Carbohydrate fractions were determined by anthrone method [30]. The soluble protein content of algal material was determined according to the method adopted by Lowry et al. [31]. Free amino acids were determined in the algal extract according to Muting and Kaiser [32]. Proline was determined according to Bates et al. [33]. Betaine and choline were determined according to Arakawa et al. [34]. Phenolic constituents were determined according to Sauvesty et al. [35].

### 2.8. Statistical Analysis

The experimental design was a random complete block, with three replications. The data were analyzed by the STATGRAPHICS (Statistical Graphics Corporation, Princeton, USA) statistical package by the* t*-test and ANOVA functions to assess significant differences among means.

## 3. Results

### 3.1. Effect of* U. lactuca* Extract on Germination Percentage and Growth Criteria of Wheat Seedlings

**Figure 2:**
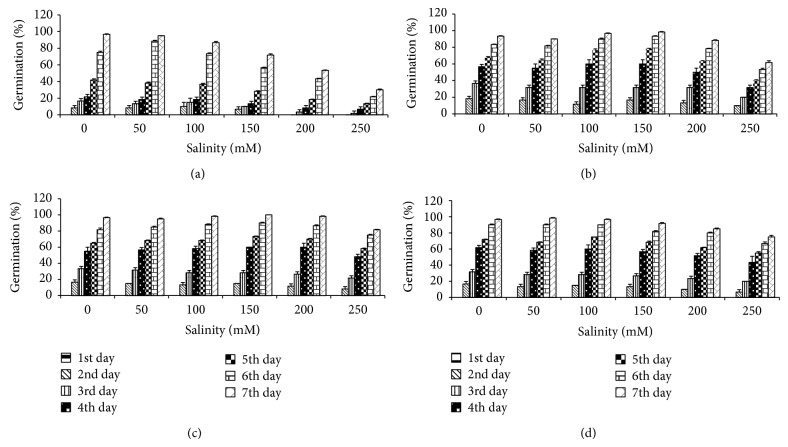
Effect of NaCl on germination percentage of* T. aestivum* grains presoaked in different concentrations of* Ulva lactuca*: (a) control, (b) algal extract 1%, (c) algal extract 5%, and (d) algal extract 10%. Data are the mean of three replicates and error bars represent the standard errors of the means.

The data in Figure 2 revealed a gradual decrease in the germination percentage of* T. aestivum* grains in response to the increment of NaCl concentration. The inhibitory effect was more obvious at the highest level of salinity (250 mM). The final germination percentage of salt stressed* T. aestivum* plants increased significantly with increasing concentration of algal extract when compared with corresponding control.

**Figure 3:**
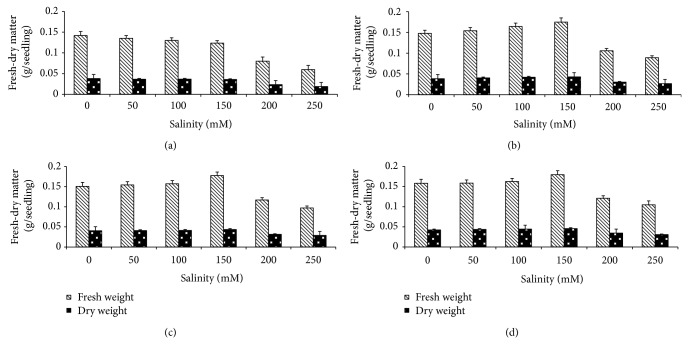
Effect of NaCl on fresh-dry matter of* T. aestivum* grains presoaked in different concentrations of* Ulva lactuca*: (a) control, (b) algal extract 1%, (c) algal extract 5%, and (d) algal extract 10%. Data are the mean of three replicates and error bars represent the standard errors of the means.

The changes of growth criteria (fresh-dry matter) of* T. aestivum* grains presoaked in different concentrations of algal extract followed by treatment with different NaCl concentrations are shown in Figure 3. Fresh-dry matter of* T. aestivum* seedlings were markedly decreased with increasing NaCl levels. Fresh-dry matter of* T. aestivum* seedlings were considerably increased with increasing algal extract concentrations from 1 to 10%. Generally, algal extract presoaked grains alleviated the adverse effects of NaCl on the growth of seedlings when compared with the corresponding treatments with NaCl.

### 3.2. Effect of* U. lactuca* Extract on Enzyme Activities and Antioxidant Contents of Wheat Seedlings

**Figure 4:**
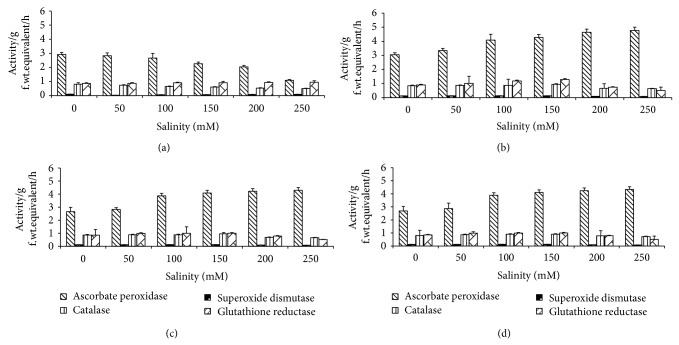
Effect of NaCl on enzyme activity of* T. aestivum* grains presoaked in different concentrations of* Ulva lactuca*: (a) control, (b) algal extract 1%, (c) algal extract 5%, and (d) algal extract 10%. Data are the mean of three replicates and error bars represent the standard errors of the means.

The results in Figure 4 showed that, the activity of enzymes APX, SOD and CAT in wheat seedling significantly decreased with increasing NaCl levels. However, the activity of GR significantly increased with increasing NaCl levels. Application of algal extract increased the activities of APX and GR at 1% algal extract above that the enzyme activities were decreased. However, the activity of SOD and CAT increased with increasing the concentration of algal extract.

**Figure 5:**
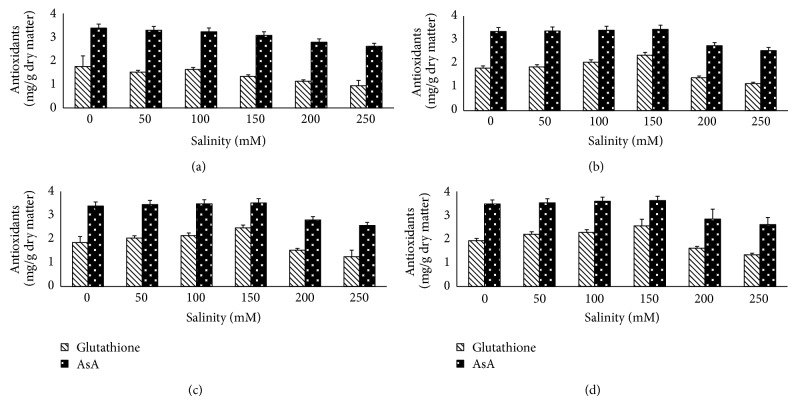
Effect of NaCl on glutathione and ascorbate of* T. aestivum* grains presoaked in different concentrations of* Ulva lactuca*: (a) control, (b) algal extract 1%, (c) algal extract 5%, and (d) algal extract 10%. Data are the mean of three replicates and error bars represent the standard errors of the means.

The glutathione and ascorbic acid contents of* T. aestivum* significantly decreased with increasing salinization levels (Figure 5). Application of algal extract increased the contents of both glutathione and ascorbic acid in* T. aestivum* seedling. Soaking of salinity stressed grains in different concentrations of algal extract increased the contents of glutathione and ascorbic acid up to 150 mM NaCl above that they were decreased compared with corresponding control.

### 3.3. Effect of* U. lactuca* Extract on Protein Profile of Wheat Seedlings

**Figure 6:**
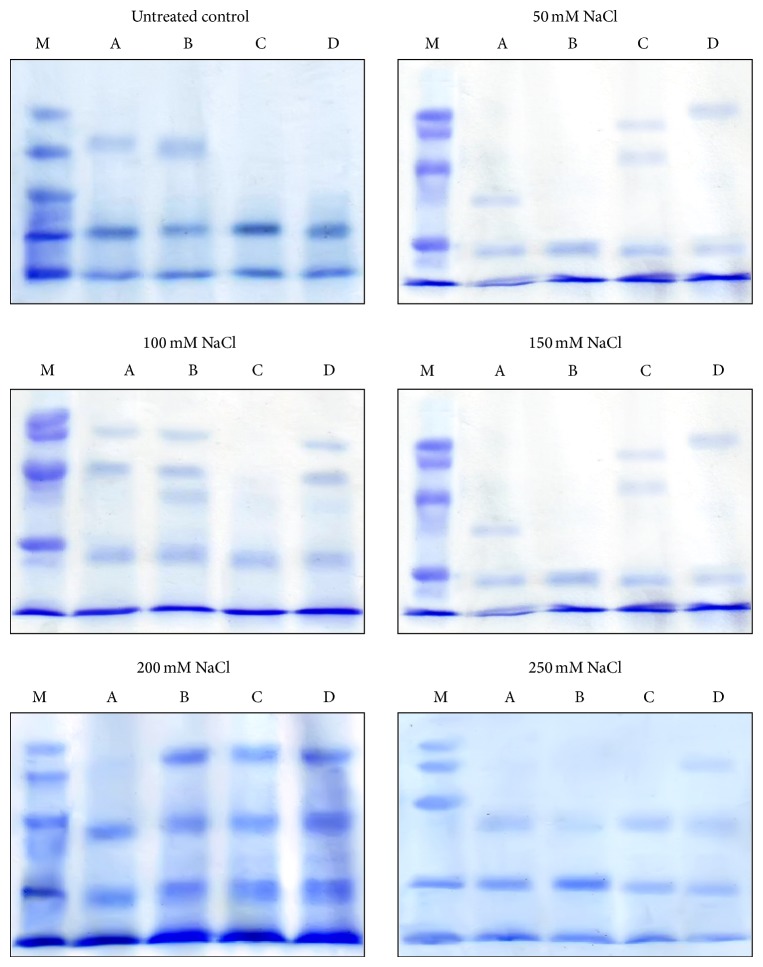
SDS-PAGE protein profile of salinity stressed* Triticum aestivum* seedlings presoaked in distilled water (A), 1%* U. lactuca* extract (B), 5%* U. lactuca* extract (C), and 10%* U. lactuca* extract (D). Protein markers (M).

SDS-PAGE protein profile of* T. aestivum* showed variations in the number of bands appearance, disappearance, and variation in the protein content, percentage, and molecular weight of each band compared with control. The SDS-PAGE is shown as an array of proteins with molecular weight ranging between 10.6 and 230 kDa (Figure 6). The total numbers of protein bands in seedlings of* T. aestivum* were 31 bands. The total band numbers have appeared in seedlings treated with different levels of salinity and/or with different concentrations of algal extract (Tables 1–4).

The electrophoretic protein pattern of salinity stressed seedling (Table 1) showed the appearance of 17 bands with molecular weight ranging between 10.6 and 230 kDa, four bands of them with *R*_*f*_ values 0.785, 0.705, 0.593, and 0.396 disappeared from protein profile of algal treated seedlings.

The protein profile of seedling presoaked in 1% algal extract and then treated with different levels of salinity is represented in Table 2. In this protein profile, 19 bands have appeared, 6 of them are considered as newly formed bands (compared with corresponding control) appeared with salinity stress 100 and 200 mM. The percent of protein content of bands with *R*_*f*_ values 0.556, 0.659, 0.761, 0.915, 0.958, and 0.988 was increased by 22.39, 66.68, 43.32, 3.77, 30.75, and 60.11%, respectively, when compared with corresponding control. On the other hand, the percent content of the bands identified by *R*_*f*_ values 0.636, 0.740, 0.795, 0.923, 0.939 and 0.945 was decreased when compared with control by 14.05, 19.31, 25.24, 6.14, 9.01, and 3.53%, respectively.

The protein profile of seedling presoaked in 5% algal extract and then treated with different concentrations of NaCl is shown in Table 3. Protein bands with *R*_*f*_ values 0.451, 0.465, 0.578, 0.585, 0.695, and 0.755 have newly appeared within protein profile of seedlings treated with 100, 150, and 200 mM NaCl. Moreover, the percent content of bands with *R*_*f*_ values 0.636, 0.795, 0.915, 0.958, and 0.988 was increased when compared with corresponding control by 17.90, 90.95, 61.76, 19.82, and 154.30%, respectively. The percent content of protein in bands with *R*_*f*_ values 0.74, 0.761, 0.923, 0.939, and 0.945 was decreased by 36.31, 27.53, 7.95, 8.17, and 17.75%, respectively.

The electrophoretic protein pattern of seedlings presoaked in 10% algal extract and treated with different levels of NaCl is represented in Table 4. Presoaking in 10% algal extract enhances the appearance of 19 bands, nine of them have newly appeared with *R*_*f*_ values 0.364, 0.451, 0.465, 0.475, 0.521, 0.585, 0.613, 0.755, and 0.842. The percent content of bands with *R*_*f*_ values 0.659, 0.915, 0.923, 0.939, 0.958, and 0.988 was increased by 93.54, 42.63, 15.33, 5.23, 21.14, and 187.11%, respectively, when compared with corresponding control. The percent content of the bands identified by *R*_*f*_ values 0.740, 0.795, and 0.945 was decreased by 12.54, 24.49, and 25.75%, respectively.

### 3.4. Biochemical Analysis of* Ulva lactuca* Biomass

The major biochemical components of* U. lactuca* (Table 5) indicated that the algal biomass contains 2.5 mg/g of protein, 0.81 mg/g of carbohydrates, and 0.58 mg/g of phenolic compounds. In addition, the algal material has high content of glutathione (0.071 mg/g), betaine (0.146 mg/g), choline (0.194 mg/g), and ascorbic acid (0.146 mg/g). Moreover, analysis of amino acids revealed that* U. lactuca* contains 1.39 and 0.78 mg/g of free amino acids and proline, respectively.

## 4. Discussion

Salinity stress is thought to result in production of reactive oxygen species (ROS) in plants causing oxidative stress [36, 37]. To ameliorate the harmful effects of salinity on plant growth, seeds presoaked in certain exogenous protectant such as osmoprotectants [38, 39], plant hormone [40], and antioxidants [41] which have been found to be effective in mitigating the salt induced damage in plant.

The data has clearly demonstrated that NaCl significantly inhibited the germination percentage at all salinity levels. The adverse effect of NaCl has been attributed to changes in osmotic potential resulting from reduced water [42]. The application of* U. lactuca* extracts had a significant stimulatory effect on seed germination of wheat plant under NaCl stress. However, the most effective treatment which had the highest germination percentage was recorded with 10% seaweed extract. These results are in harmony with those reported by Hemmat [43] and Abd El-Baky et al. [44].

*U. lactuca* extract led to a marked increase in growth of wheat seedling under salinity stress. This increment could be due to the presence of some biochemical compounds in* U. lactuca* extract such as ascorbic acid, proline, and glutathione (Table 5) that might be required for the induction of antioxidant enzymes responsible for decreasing ROS levels in the salinity stressed plant [38, 39, 45].

A correlation between antioxidants capacity and NaCl tolerance has been demonstrated in several plant species. The present investigation was therefore undertaken to study the effect of NaCl on ascorbate and glutathione contents. The results showed that presoaking of grains in algal extract significantly increased the seedlings glutathione and ascorbic acid under saline conditions when compared with water presoaked grains. These results are in accordance with the results obtained by Akladious and Abbas [46] and Hemida et al. [45].

Antioxidative enzymes are the first response mechanism against salinity stress. As such, their activity profiles are important in the evaluation of tolerance mechanisms. The results showed significantly decrease in the activities of APX, SOD, and CAT in salinity stressed seedlings. Presoaking of grains in different concentrations of* U. lactuca* extract increased the activities of APX, SOD, and CAT with increasing the concentration of algal extract. Increment of the enzyme activities of salinity stressed seedlings presoaked in different concentrations of algal extract could be attributed to the presence of antioxidative compounds such as ascorbic acid (0.146 mg/g), proline (0.78 mg/g), betaine (0.146 mg/g), and glutathione (0.071 mg/g) in* U. lactuca* extract [45, 47, 48].

Under stress conditions, total protein synthesis usually decreases in plant cells [49], but some proteins that are specifically responding to stress (stress induced proteins) are induced in many plants [50]. Although the expression and function of such proteins are nuclear, it is suggested that there is a relationship between some forms of plant stress adaptation and the expression of stress induced proteins [50, 51].

One possible explanation for bands which have disappeared in protein pattern of salinity stressed seedlings and newly formed in protein pattern of algal presoaked seedlings is that the gene(s) responsible for certain proteins had not been completely suppressed, but inhibited as the result of stress [52, 53]. Presoaking of grains with* U. lactuca* extracts alleviates salinity stress and induces the formation of these proteins in seedlings cells, changing number and percentage of bands in protein pattern of algal presoaked seedlings.

## 5. Conclusions

To our knowledge, this is the first study for the application of* Ulva lactuca* extract to overcome the negative effect of NaCl on wheat seedlings. Our results confirmed that pretreatment of grains increases the germination percentage, seedling growth, and antioxidant content and induces the formation of new bands in the protein pattern. Overall, it can be concluded that algal extract could improve physiological properties of wheat seedlings under salt stress conditions. Hence, work in this regard should continue to characterize the specific bioactive materials in* U. lactuca* extract responsible for the alleviation of salinity stress in order to evaluate its efficiency to adverse effects of salinity on growth of wheat plants.

## Figures and Tables

**Table 1:** Electrophoretic protein pattern of salinity stressed *Triticum aestivum* seedlings presoaked in distilled water.

M. Wt (KDa)	*R*_*f*_	Salinity levels											
		0 mM		50 mM		100 mM		150 mM		200 mM		250 mM	
		% Content	Band %	% Content	Band %	% Content	Band %	% Content	Band %	% Content	Band %	% Content	Band %
230.1	0.396					100	15.39^a^						
142.39	0.412					100	15.81						
79.71	0.556	100	14.2										
49.01	0.593									100	24.06^a^		
44.72	0.636											100	18.74
42.26	0.659					100	18.68						
34.89	0.705							100	17.13^a^				
22.85	0.74	100	45.51										
22.50	0.761											100	38.5
20.55	0.785									100	30.18^a^		
20.12	0.795							100	36.07				
12.71	0.915							100	46.8				
11.92	0.923	100	40.29										
11.35	0.939					100	50.12						
11.33	0.945											100	42.75
11.21	0.958									100	45.76		
10.63	0.988			100	100								

^a^Bands disappeared from protein profile of *U. lactuca* treated seedlings.

**Table 2:** Electrophoretic protein pattern of salinity stressed *Triticum aestivum *seedlings presoaked in 1% *Ulva lactuca* extract.

M. Wt (KDa)	*R*_*f*_	Salinity levels											
		0 mM		50 mM		100 mM		150 mM		200 mM		250 mM	
		% Content	Band %	% Content	Band %	% Content	Band %	% Content	Band %	% Content	Band %	% Content	Band %
215.04	0.364					0	14.2^b^						
132.44	0.431					0	9.13^b^						
125.82	0.451									0	16.6^b^		
98.09	0.494					0	18.2^b^						
79.72	0.556	122.39	18.91										
52.73	0.585									0	25.1^b^		
44.72	0.636											85.95	14.32
42.26	0.659					166.68	23.72						
22.85	0.74	80.69	39.95										
22.65	0.755									0	23.8^b^		
22.50	0.761											143.32	49.04
20.12	0.795							74.76	35.7				
12.71	0.915							103.77	64.3				
11.92	0.923	93.86	41.14										
11.35	0.939					90.99	34.72						
11.33	0.945											96.47	36.65
11.21	0.958									130.75	34.55		
10.63	0.988			160.11	100								

^b^Newly formed bands in protein profile of *U. lactuca* treated seedlings.

**Table 3:** Electrophoretic protein pattern of salinity stressed *Triticum aestivum *seedlings presoaked in 5% *Ulva lactuca* extract.

M. Wt (KDa)	*R*_*f*_	Salinity levels											
		0 mM		50 mM		100 mM		150 mM		200 mM		250 mM	
		% Content	Band %	% Content	Band %	% Content	Band %	% Content	Band %	% Content	Band %	% Content	Band %
125.82	0.451									0	13.9^b^		
117.84	0.465							0	10.2^b^				
65.76	0.578							0	9.0^ b^				
52.73	0.585									0	24.1^b^		
44.72	0.636											117.9	25.95
35.83	0.695					0	29.2^b^						
22.85	0.74	63.69	43.87										
22.65	0.755									0	26.2^b^		
22.50	0.761											72.47	32.76
20.12	0.795							190.95	38.5				
12.71	0.915							161.76	42.32				
11.92	0.923	92.05	56.13										
11.35	0.939					91.83	70.84						
11.33	0.945											82.25	41.29
11.21	0.958									119.82	35.64		
10.63	0.988			154.3	100								

^b^Newly formed bands in protein profile of *U. lactuca* treated seedlings.

**Table 4:** Electrophoretic protein pattern of salinity stressed *Triticum aestivum *seedlings presoaked in 10% *Ulva lactuca* extract.

M. Wt (KDa)	*R*_*f*_	Salinity levels											
		0 mM		50 mM		100 mM		150 mM		200 mM		250 mM	
		% Content	Band %	% Content	Band %	% Content	Band %	% Content	Band %	% Content	Band %	% Content	Band %
215.04	0.364					0	13.4^b^						
142.40	0.412					65.25	7.47						
125.82	0.451									0	17^b^		
117.84	0.465							0	20.9^b^				
104.36	0.475					0	14.8^b^						
94.99	0.521											0	14.3^b^
52.73	0.585									0	23.9^b^		
46.88	0.613											0	25.2^b^
42.26	0.659					193.54	26.17						
22.85	0.74	87.46	46.14										
22.65	0.755									0	28.1^b^		
20.12	0.795							75.51	22.93				
17.82	0.842											0	28.5^b^
12.71	0.915							142.63	56.19				
11.92	0.923	115.33	53.86										
11.35	0.939					105.23	38.17						
11.33	0.945											74.25	32.06
11.21	0.958									121.14	31		
10.63	0.988			287.11	100								

^b^Newly formed bands in protein profile of *U. lactuca* treated seedlings.

**Table 5:** Some biochemical analyses of marine alga* Ulva lactuca*.

Protein (mg/g)	2.5 ± 0.032	Glutathione (mg/g)	0.071 ± 0.001
Carbohydrate (mg/g)		Ascorbic acid (mg/g)	0.146 ± 0.006
Soluble	0.58 ± 0.005	Betaine (mg/g)	0.146 ± 0.003
Insoluble	0.23 ± 0.005	Choline (mg/g)	0.194 ± 0.003
Phenolic compound (mg/g)		Amino acids (mg/g)	
Glychone	0.35 ± 0.008	Free amino acids	1.39 ± 0.003
Aglychone	0.23 ± 0.007	Proline	0.78 ± 0.005

Values are expressed as mean ± standard error of three replicates.
